# Evidence-based control of canine rabies: a critical review of population density reduction

**DOI:** 10.1111/j.1365-2656.2012.02033.x

**Published:** 2012-09-24

**Authors:** Michelle K Morters, Olivier Restif, Katie Hampson, Sarah Cleaveland, James L N Wood, Andrew J K Conlan

**Affiliations:** 1 Department of Veterinary Medicine, University of CambridgeCambridge, UK; 2 Institute of Biodiversity, Animal Health and Comparative Medicine, University of GlasgowGlasgow, UK

**Keywords:** culling, density, dog, sterilisation, vaccination

## Abstract

1. Control measures for canine rabies include vaccination and reducing population density through culling or sterilization.
2. Despite the evidence that culling fails to control canine rabies, efforts to reduce canine population density continue in many parts of the world.
3. The rationale for reducing population density is that rabies transmission is density-dependent, with disease incidence increasing directly with host density. This may be based, in part, on an incomplete interpretation of historical field data for wildlife, with important implications for disease control in dog populations. Here, we examine historical and more recent field data, in the context of host ecology and epidemic theory, to understand better the role of density in rabies transmission and the reasons why culling fails to control rabies.
4. We conclude that the relationship between host density, disease incidence and other factors is complex and may differ between species. This highlights the difficulties of interpreting field data and the constraints of extrapolations between species, particularly in terms of control policies.
5. We also propose that the complex interactions between dogs and people may render culling of free-roaming dogs ineffective irrespective of the relationship between host density and disease incidence.
6. We conclude that vaccination is the most effective means to control rabies in all species.

## Introduction

Canine-mediated rabies is a serious zoonosis causing an estimated 55 000 human deaths per year (Knobel *et al*. 2005). Mortality from rabies is highest in developing communities in Africa and Asia where domestic dogs are predominately free-roaming (Ezeokoli & Umoh 1987; Butler & Bingham 2000; Kitala *et al*. 2002; Kayali *et al*. 2003; Windiyaningsih *et al*. 2004; Kasempimolporn, Jitapunkul & Sitprija 2008). Social, economic and political factors contribute to the inadequate control of rabies in domestic dog populations (WHO 2004), accentuated by an incomplete understanding of disease dynamics. Knowledge of the factors that drive the transmission of rabies is needed for the development of effective, sustainable disease control measures.

Two main methods are used to control canine rabies: vaccination (Cleaveland *et al*. 2003; WHO 2004; Schneider *et al*. 2005; Cleaveland *et al*. 2006) and measures aiming to reduce dog population density, usually by culling (i.e. the widespread killing of dogs regardless of infection status) (Beran & Frith 1988; Windiyaningsih *et al*. 2004) but also by sterilization (WHO 2004; Reece & Chawla 2006). Dog vaccinations are often undertaken as annual campaigns that aim to achieve 70% coverage (WHO 2004). This target coverage is supported by empirical evidence and theory, which indicates that a 70% coverage achieved during campaigns should maintain population immunity above the critical levels (25–40%) required to interrupt rabies transmission (Coleman & Dye 1996; Cleaveland *et al*. 2003; Hampson *et al*. 2009). This additional coverage above the critical level compensates for the loss in coverage arising from an increase in susceptible and loss of immune dogs through demographic and immunological processes (Hampson *et al*. 2009). Culling of dogs is also used, alone or with vaccination (Kaplan, Goor & Tierkel 1954; Larghi *et al*. 1988), based on the assumption that a physical reduction in the number of dogs must reduce the incidence of rabies, despite evidence suggesting that it is ineffective (Beran & Frith 1988; WHO 2004; Windiyaningsih *et al*. 2004). Culling is still used, partly as a visible response to public concerns about rabies. It is also perceived to be easier to implement than annual vaccination of 70% of dogs, particularly if many are free-roaming and poorly socialized, and in areas where veterinarians and animal health workers have relatively little experience or confidence in handling dogs. In some areas, sterilizations are carried out together with vaccinations, on the basis that this is a more humane and culturally acceptable approach to reducing dog population density.

The theoretical basis for rabies control measures involving culling or sterilization is the assumption that rates of transmission are density-dependent (Anderson *et al*. 1981; Wandeler *et al*. 1988; Cleaveland 1998; Hampson *et al*. 2007). This scaling of transmission rates occurs if the rate of encounters between susceptible and infectious individuals increases with host population density. Under this assumption, we expect that disease incidence will also increase with host density, as will the basic reproductive number (R_0_) that characterizes the maximum reproductive potential of a pathogen. R_0_ is defined as the average number of secondary infections produced when one infected individual is introduced into a wholly susceptible population (Anderson & May 1991). For an epidemic to spread, R_0_ must, by definition, be >1. Hence, under density-dependent transmission, there will exist a threshold density below which disease cannot invade a population. This contrasts with frequency-dependent disease transmission where the rate of contact and subsequent rates of transmission are assumed to be independent of host density and a threshold density for invasion does not exist (Begon *et al*. 2002; Lloyd-Smith *et al*. 2005).

Under either frequency- or density-dependent transmission, vaccination equally reduces both the number and proportion of susceptible individuals in a host population, and thus, the opportunities for transmission to occur. Therefore, the assumption that rabies transmission is density-dependent has little consequence for the efficiency of vaccination programmes. Conversely, the assumption is of critical importance with regard to control measures that aim to reduce dog population density. The net impact of culling and sterilization on subsequent rates of rabies transmission depends on the degree to which transmission scales with population density. Under the assumption of frequency dependence, density reduction will have no impact on the rate of transmission. Conversely, when transmission is density-dependent, there will be a threshold for disease invasion, and density reduction alone has the potential to achieve disease eradication. However, stochastic effects and antagonistic biological processes may complicate these simple relationships.

Establishing the relationships between host density, disease incidence and other processes is therefore not only important for refinement of epidemiological models for rabies transmission, but also has serious practical implications for the utility of density reduction in controlling rabies. In this study, we review current understanding of the role of density and other factors in rabies transmission in dogs to encourage reappraisal of the most appropriate and effective means of rabies control. Within the literature, and during the development of policy, extrapolations are often made between species, in particular between wildlife and domestic dogs. We therefore extend our review to rabies transmission in wildlife and highlight the differences and similarities with dog populations. We also compare the utility of various lines of evidence between species. This discussion will focus on fox rabies in particular, as empirical data on the local transmission of wildlife rabies are largely confined to this host species.

## Evidence for density-dependent transmission of rabies

It is difficult to determine the direct relationship between disease incidence, host density and transmission under field conditions, particularly for wildlife given their inaccessibility (Wandeler *et al*. 1974b; Macdonald & Voigt 1985; Beyer *et al*. 2010). Consequently, we are left with interpreting indirect and somewhat conflicting evidence regarding the role of density in rabies transmission in wildlife and dogs. In this section, we examine four key lines of evidence about the functional forms of rabies transmission.

### Cycles in Disease Incidence

Cycles in disease incidence have motivated some of the most effective applications of population modelling in ecology (Anderson & May 1991; Begon, Harper & Townsend 1996). Mathematical models can explore how different biological hypotheses relate to the expected amplitude and period of cycles, providing insights into the drivers of transmission. Perhaps, the most successful examples of this have been in the study of childhood infectious diseases (Earn *et al*. 2000; Altizer *et al*. 2006) where detailed historical records have allowed the application of sophisticated methods of statistical inference (Bjornstad, Finkenstadt & Grenfell 2002; Grenfell, Bjornstad & Finkenstadt 2002). However, even in the absence of detailed data, models can provide useful insights simply through the ability of a given mechanism to generate periodic dynamics.

Cycles have been observed for wildlife (Friend 1968; Bogel *et al*. 1974; Childs *et al*. 2000; Courtin *et al*. 2000; MacInnes *et al*. 2001) and canine rabies (Ernst & Fabrega 1989; Bingham *et al*. 1999a; Widdowson *et al*. 2002; Hampson *et al*. 2007), although periodicity in incidence is not a consistent finding (Macdonald & Voigt 1985; Zinsstag *et al*. 2009). The mechanistic driver of these cycles is widely assumed to be the interaction of density-dependent transmission, rabies-induced mortality and other demographic processes (Bogel *et al*. 1974; Steck & Wandeler 1980; Anderson *et al*. 1981; Childs *et al*. 2000; Hampson *et al*. 2007). However, it is important to determine wether this assumption is correct given its implications for culling.

Deterministic compartmental models have been used to describe rabies dynamics in wildlife (Anderson *et al*. 1981; Coyne, Smith & McAllister 1989) and domestic dogs (Cleaveland & Dye 1995; Coleman & Dye 1996; Kitala *et al*. 2002; Hampson *et al*. 2007; Carroll *et al*. 2010). These models assume random mixing, neglecting the spatial and social heterogeneity that exists in real populations. Within such ‘well-mixed’ models, frequency-dependent transmission of fatal diseases inevitably leads to rapid die-out of the host population (Keeling & Rohani 2008). Under frequency dependence, the average reproductive potential of the pathogen is unchanged during the spread of an epidemic. With no mechanism to arrest the spread of disease, transmission continues and the host and parasite populations go extinct. In contrast, under the assumption of density-dependent transmission, epidemics will subside when the host density falls below the invasion threshold (where R_0_ = 1). The time delay between epidemic peak and replenishment of the host population generates damped epidemic cycles through delayed density dependence. The assumption of density-dependent transmission is therefore the most parsimonious mechanism by which stable epidemic cycles for rabies can be supported within deterministic random mixing models. However, in structured populations, epidemic cycles may be generated by alternative mechanisms even when the transmission rate is frequency-dependent.

Age structure is one such potential mechanism. Attack rates for rabies appear to vary considerably with age, with reported incidence in foxes in Europe (Wandeler *et al*. 1974b) and raccoons in Ontario (Rosatte *et al*. 2006) concentrated within adult age classes. Within an age-structured model, the net reproductive ratio of rabies will not only depend on the rates of transmission, but also on the age distribution in the population (Anderson & May 1991). If the basic reproductive ratio is only above unity for a core group of high-risk individuals, the epidemic can recede when this core group is exhausted. The delay between depletion of the core group and replenishment through births can generate cycles in incidence that may be sustained by seasonal birth pulses (Davis & Wood 1959; Lloyd *et al*. 1976).

Deterministic thresholds are not the only possible mechanism by which endemic coexistence of rabies could be maintained within frequency-dependent transmission models. An important limitation of deterministic models is that they do not account for the probability of local extinction of disease following an epidemic. In areas where rabies in foxes is not actively controlled, 3–4 yearly cycles in incidence are observed at regional levels [around 1000 km^2^ in Europe and at the county level in Canada] (Johnston & Beauregard 1969; Bogel *et al*. 1974) and are out of phase between regions (Johnston & Beauregard 1969; Bogel *et al*. 1974; Macdonald & Voigt 1985). Epidemics have been associated with considerable reductions in host populations by up to 50% (Bogel *et al*. 1974). This reduction in the density of the host species within a region and the corresponding reduction in the instantaneous numbers of infective individuals will increase the chances of rabies becoming locally extinct before the host population is exhausted. Stochastic population thresholds for persistence of rabies can exist irrespective of the mode of transmission (Lloyd-Smith *et al*. 2005). Stochastic extinction and re-introduction of rabies following the local restructuring of host populations (Steck & Wandeler 1980; Anderson *et al*. 1981; Macdonald & Voigt 1985), consistent with metapopulation dynamics, are also viable alternative mechanisms to generate these dynamics.

In conclusion, cycles in rabies incidence observed in wildlife could be supported by density- or frequency-dependent transmission when stochasticity and the heterogeneous structure of real populations are accounted for.

Although deterministic density-dependent models have been used to describe rabies dynamics in domestic dogs, reactive vaccination can also drive cycles in incidence (Hampson *et al*. 2007). For example, in Zimbabwe between 1950 and 1995, the amplitude and interval of peaks in rabies varied (from 75 to 350 cases per year and interepidemic periods from 4 to 20 years) with the level of vaccination delivered during national vaccination campaigns (Bingham *et al*. 1999a). These observations provide little insight into the processes driving local disease dynamics for dogs. Rather, other evidence for the functional forms of transmission of canine rabies will be considered in the next sections.

### The Relationship between R_0_ and Host Density

As discussed above, R_0_ is expected to increase with density for density-dependent transmission and remains constant irrespective of density for frequency-dependent transmission. R_0_ may be estimated from the (exponential) rate of growth early in an epidemic prior to significant susceptible depletion or implementation of control measures (Heffernan, Smith & Wahl 2005; Wallinga & Lipsitch 2007). Using this method, Hampson *et al*. (2009) obtained estimates of R_0_ for canine rabies, across a wide geographical range, of between 1·05 and 1·72. The range of these estimates is similar to the statistical uncertainty in simulated epidemics when the biting behaviour of rabid dogs is accounted for. Dog population densities were reported for only four of these locations, ranging from 1·36 dogs km^−2^ in rural Tanzania to 110 unrestricted dogs per km^2^ in urban Mexico. However, other locations cited in the study are likely to represent even higher densities, with the highest reported density in the general literature being 2388 dogs km^−2^ in Guayquil, Ecuador (Beran & Frith 1988). The absence of any correlation between R_0_ and host density across such a large range of densities is consistent with earlier studies (Coleman & Dye 1996; Kitala *et al*. 2002) and suggests that if a relationship between transmission and dog density does exist, it must be quite weak.

Equivalent data are not available for wildlife. Compared to canine rabies, incidence records generally have a lower temporal resolution (typically quarterly or annually) (Macdonald & Voigt 1985; Rhodes *et al*. 1998; Bingham *et al*. 1999b; Rosatte *et al*. 2006), and the ranges of host densities are narrower: 0·8–1·2 jackals km^−2^ during the breeding season on commercial farmland in Zimbabwe (Rhodes *et al*. 1998), 5·4–9·1 racoons km^−2^ (averaged over a 4 year period) for rural Ontario (Rosatte *et al*. 2007) and 0·5–1·8 adult foxes km^−2^ in central Europe (Lloyd *et al*. 1976).

This apparent lack of relationship between R_0_ and host density is most consistent with frequency-dependent transmission. However, as previously discussed, random mixing models with frequency-dependent transmission of rabies predict host extinction as soon as R_0_ exceeds unity. This prediction is inconsistent with the very low attack rates reported for canine rabies compared to wildlife rabies and with the absence of large declines in population densities from rabies-induced mortality (Hampson *et al*. 2007). Estimates of the incidence, or average monthly attack rates, are typically below 0·5% and rarely exceed 2% (Waltner-Toews *et al*. 1990; Windiyaningsih *et al*. 2004; Zinsstag *et al*. 2009; Tenzin *et al*. 2010; Putra *et al*. 2011; Tenzin *et al*. 2011).

This incongruity between attack rates and the apparent scaling of R_0_ may be resolved by considering a more complex relationship between rabies dynamics in dogs and anthropogenic factors than has previously been assumed. Suspect rabid and in-contact dogs are often identified and killed swiftly by the community (Hampson *et al*. 2007, 2009), a practice hereafter referred to as ‘selective removal’. This reduces the effective infectious period in dogs (Hampson *et al*. 2009) and could contribute to the relatively lower incidence as compared to wildlife. The selective removal of infectious and in-contact dogs was thought to have contributed to the control of rabies in eastern Bhutan (Tenzin *et al*. 2011) and the United Kingdom (Pastoret & Brochier 1998). Indeed, euthanasia (WSPA 2012) of infected dogs is advocated to control rabies (WHO 2004). Such behavioural responses to the spread of epidemics are rarely considered in epidemiological models (Ferguson 2007; Funk *et al*. 2009) but are likely to play a particularly important role in disease transmission within owned, and managed, populations. Selective removal may conceal the existence of density-dependent transmission processes if the rate of intervention also scales with density.

We thus hypothesize that selective removal itself might be density-dependent for several reasons. First, rabid dogs may be more quickly spotted and selectively removed from areas with more people present. Second, given that most dogs are owned (WHO & WSPA 1990; Cleaveland & Dye 1995; Butler & Bingham 2000; Windiyaningsih *et al*. 2004), dog and human population densities are expected to correlate (Oboegbulem & Nwakonobi 1989; Matter *et al*. 1998; Butler & Bingham 2000). Finally, other anthropogenic factors that may interfere with contact processes, such as traffic or urban infrastructure, are also likely to scale with human and dog density. Therefore, the effective infectious period, as reduced by selective removal, could scale inversely with human, and thus dog, population density. The estimates of R_0_ discussed above are conditional on the assumption of a fixed infectious period. Any systematic variation in the infectious period with population density could counteract the impact of density-dependent contact rates and result in R_0_ appearing density-independent. Under this hypothesis, density-dependent transmission could not be ruled out unequivocally for canine rabies.

As a final consideration, stochastic fade-out is expected with low attack rates. However, rabies often appears to persist in dog populations. This may be because selective removal and stochastic processes are offset by the continual translocation of dogs (some of them infected) by people (Beran & Frith 1988; Denduangboripant *et al*. 2005; Coetzee & Nel 2007; Kasempimolporn, Jitapunkul & Sitprija 2008; Zinsstag *et al*. 2009) consistent with metapopulation dynamics (Hanski & Gaggiotti 2004; Beyer *et al*. 2010). In conclusion, more intensive study of the mechanisms underlying rabies transmission and persistence in domestic dog populations is warranted to understand these empirical patterns.

### Thresholds for Invasion and Increasing Incidence with Population Density

The existence of a threshold in host population density below which infection cannot spread (i.e. where R_0_ < 1) would be direct evidence in support of density-dependent transmission. Such invasion thresholds in wildlife and domestic dog populations have been proposed based on a limited number of studies that compared disease incidence between different geographical locations with different host densities (Steck & Wandeler 1980; Beran & Frith 1988; Cleaveland & Dye 1995). However, as discussed below, it is not possible to establish the relationship between host density and disease incidence based on these data.

Threshold densities for invasion have been suggested to occur where canine rabies is observed to change from sporadic disease at lower densities to persistence at higher densities (Beran & Frith 1988; Cleaveland & Dye 1995). However, these observations could also be explained by increased stochastic fade-out of disease at lower densities where there are lower numbers of infected dogs. In general, the probability of stochastic fade-out will decrease with an increase in R_0_ or in the number of infected individuals (Lloyd-Smith *et al*. 2005). This effect may be particularly relevant to dogs where more infected individuals may be introduced into larger or more dense populations by people (Denduangboripant *et al*. 2005; Kasempimolporn, Jitapunkul & Sitprija 2008; Zinsstag *et al*. 2009). Consequently, the probability of stochastic fade-out is predicted to decrease with an increase in population size or density. Even when R_0_ is invariant between populations of different sizes or densities, stochastic effects may give the impression of a deterministic threshold for invasion where one does not exist. This is particularly likely when R_0_ is low. Should a deterministic threshold for invasion exists, it may be obscured by these processes and be lower than estimated empirically.

The key data used to support the existence of a threshold density in foxes are expressed in terms of the hunting indicator of population density (HIPD) (Steck & Wandeler 1980). HIPD is an indirect estimate of density, with well-known biases (Wandeler 1980; Macdonald & Voigt 1985). However, there are two specific issues with the use of these data to support a threshold density for fox rabies. First, HIPD estimates below the purported threshold density for invasion were not recorded, thus precluding any conclusion of an invasion threshold. Second, the observed positive correlation between the annual number of animal rabies cases per km^2^ per year and the HIPD has been wrongly interpreted as evidence for density-dependent transmission. Assuming the HIPD correlates with host density, such a relationship would be expected whether transmission depends on fox density or not. Determining the mode of transmission would require an evaluation of disease incidence as a proportion of the total population size or density (Rothman, Greenland & Lash 2008), which cannot be inferred from HIPD.

### Impacts of Density Reduction

Density reduction, particularly culling (i.e. the widespread killing of hosts regardless of infection status), has been undertaken to reduce the incidence of rabies and therefore eliminate the disease on the basis that transmission is density-dependent. As previously discussed, the assumption of density dependence originates from the interpretation of cycles in wildlife rabies and thresholds for the invasion for foxes and dogs. However, the fact that culling has failed to achieve sustained control of rabies in wildlife and dogs (Kaplan, Goor & Tierkel 1954; Anderson *et al*. 1981; Macdonald & Voigt 1985; Anderson 1986; Beran & Frith 1988; WHO 2004; Windiyaningsih *et al*. 2004; Cleaveland *et al*. 2006) may be the best evidence that a simple relationship between disease incidence and host population density does not exist for rabies. We now discuss evidence from culling programmes (dogs and wildlife) followed by more limited evidence on sterilization campaigns.

#### Culling

Culling has been shown to be ineffective in controlling rabies in all host species. Rabies persisted in foxes in New York State despite ‘concentrated reduction campaigns’ following an outbreak in 1945, while simultaneous vaccination of dogs in the State eliminated rabies from this species (Friend 1968). Similarly, in Denmark in 1964, culling did not prevent rabies outbreaks in foxes; however, rabies did not occur where dogs in the same region had been vaccinated (Muller 1966, 1971). In response to a rabies outbreak in 1997, nearly 300 000 dogs, approximately half of the population estimated at the start of the outbreak, were culled in Flores, Indonesia over a period of 4 years. However, in 2004, rabies was still endemic although the total dog population was still considerably reduced (Windiyaningsih *et al*. 2004). Culling failed to control canine rabies in Korea (Lee *et al*. 2001) and Israel (Kaplan, Goor & Tierkel 1954), whereas subsequent vaccination in both countries controlled the disease.

Culling has been used to control ongoing outbreaks and to prevent the invasion of rabies in foxes. Declines in rabies cases have followed outbreaks irrespective of active culling (Bogel *et al*. 1974), with stochastic extinction expected (Anderson *et al*. 1981) particularly where disease-induced mortality is substantial (Bogel *et al*. 1974). Within a given area, culling might be expected to amplify these processes, increasing the probability of stochastic extinction regardless of density dependence. Indeed, rabies appeared to die-out in some areas where fox dens were gassed (Wandeler *et al*. 1974b). However, the limited data available are unclear regarding how culling interacts with disease-induced mortality during an epidemic and how it may change disease dynamics (Wandeler *et al*. 1974b). Other processes may also counter the effect of density reduction on disease incidence. Examples include social perturbations, as demonstrated in badger populations (Woodroofe *et al*. 2006a,b), and interactions between the level of culling, age structure (Bolzoni, Real & De Leo 2007) and demographic processes (Choisy & Rohani 2006).

Culling has also failed to prevent outbreaks of rabies in foxes in previously unaffected areas or the recurrence of the disease in areas where it had died-out, as observed in southern Denmark (Muller 1971). Where density-dependent transmission has been assumed, invasion thresholds are reported to vary and to be low (i.e. <1 fox km^−2^ in Europe and <0·4 foxes km^−2^ in Ontario). Thus, even if transmission were density-dependent, reductions in density to below an invasion threshold may not be achievable practically or be sustainable (Wandeler *et al*. 1974a; Anderson *et al*. 1981).

Culling has generally failed to eliminate outbreaks of rabies in dogs. In our review of the scaling of rabies transmission rates with density (in the previous sections), we have found no conclusive evidence to support either the frequency-dependent or density-dependent assumption for canine rabies. We are therefore unable to unequivocally conclude that the ineffectiveness of culling is because transmission is frequency-dependent. An alternative explanation is that reductions in densities to below invasion thresholds are not achievable practically. Canine rabies can circulate where densities are as low as 1·36 dogs km^−2^ (Hampson 2009), which is substantially lower than the densities reported for most free-roaming dog populations. Under the assumption of density-dependent contact rates, culling and vaccination should have similar impacts on disease incidence. Thus, given estimated values of R_0_ < 2, control should be achieved by culling at most half the population. Yet, in Flores, Indonesia, rabies persisted after this level of culling was achieved (Windiyaningsih *et al*. 2004). More generally, the stochastic persistence of canine rabies despite low attack rates and considerable density reduction is interesting irrespective of the mode of transmission.

The fact that rabies often persists despite culling may be a function of human factors. The continual translocation of dogs (some infected) with people (Beran & Frith 1988; Denduangboripant *et al*. 2005; Coetzee & Nel 2007; Kasempimolporn, Jitapunkul & Sitprija 2008; Zinsstag *et al*. 2009) may offset the selective removal of infectious and in-contact dogs and stochastic extinctions. Where culling occurs simultaneously, translocation may also offset any reductions in the incidence of rabies. In addition, translocation may be exacerbated in response to culling campaigns. For example, within a few days of a village-wide cull in Kelusa, Bali, where rabies had not occurred previously, two residents brought in unvaccinated, potentially infected puppies from outside the village to replace their culled, vaccinated adult dogs. As attack rates are typically very low, culling predominately removes healthy dogs, and some of these may be vaccinated and hence unlikely to become infected. Other compensatory mechanisms may also offset reductions in host density. These include concomitant reductions in mortality from reduced competition for food (although the actual intensity of competition in free-roaming dogs is unknown), reductions in the dumping of surplus puppies/unwanted dogs and improved care of dogs. To address these issues, we are currently investigating the effects of human behaviour in response to culling on dog population dynamics and disease transmission in Kelusa.

The ethics of culling healthy, free-roaming animals in conjunction with vaccination programmes are also debatable. Raccoons have been culled on Wolfe Island, Ontario, as a means to reduce the number of animals that needed to be trapped and vaccinated (Rosatte *et al*. 2007). The same justification may be extended to dogs, and a variable degree of culling of free-roaming dogs, historically regarded as ‘strays’, has often been undertaken alongside mass vaccination programs (Wells 1954; Cheuk 1969; Larghi *et al*. 1988; Ernst & Fabrega 1989). However, despite appearances, the vast majority of free-roaming dogs in most societies globally are owned (WHO & WSPA 1990; Cleaveland & Dye 1995; Butler & Bingham 2000; Windiyaningsih *et al*. 2004) and in reasonable health. Not only are these dogs more accessible to vaccination than commonly recognized, but culling healthy animals can result in unintended negative consequences on both animal welfare and disease control.

#### Sterilization

The use of immunological and chemical sterilization has been modelled for the control of rabies in wildlife and in dogs (Suppo *et al*. 2000; Smith & Cheeseman 2002; Carroll *et al*. 2010). However, only surgical sterilization has been used in dogs under field conditions. Sterilizations are usually carried out by nongovernmental organizations and local authorities, which aim to vaccinate and simultaneously sterilize at least 70% of the dog population (Totton 2009). Limited data suggest that these programs reduce the incidence of rabies and may stabilize or gradually reduce population density over time-scales of several years (Reece & Chawla 2006; Totton 2009; Totton *et al*. 2010). However, the respective impacts of vaccination and sterilizations have not been assessed. Reductions in population density may plausibly reduce the number of dogs that require vaccination, although timely reductions in density may be constrained by resources and population dynamics (Hemachudha 2005). As with culling, the demand for dogs by communities may result in an increase in dog importation where local supply has been reduced by sterilization. Thus, we are studying the effect of human behaviour in response to sterilization on dog population dynamics and disease transmission in Antiga, Bali.

## Conclusion

There is still considerable uncertainty surrounding the role of density in the transmission of rabies in animal host species. Density has been assumed to be the key factor that drives transmission, with important implications for the use of population reduction as a means to control rabies. However, it is evident that the relationship between host density, disease incidence and other factors is complex and varies between species. Further research to determine the factors that drive rabies transmission would not only enhance development of epidemiological models but also inform the development of effective, sustainable disease control measures.

Determining the effect of density in the transmission of rabies in wildlife hosts is constrained by the lack of high-resolution data exhibiting sufficient variability in both disease incidence and host densities. We have discussed how cycles in the incidence of rabies in foxes and raccoons can occur under either frequency- or density-dependent transmission, and how both model structures could account for the failure of culling to control rabies.

Although still limited, better quality data for dogs suggest a more complicated relationship between contact rates and host density. The evidence indicates that not only is reducing dog density ineffective at controlling rabies, but culling in particular often has unintended negative consequences. We advocate more systematic investigation of the human factors that could affect the dynamics of rabies in dogs, to understand possible contrasts with the situation in wildlife.

In contrast to culling, vaccination programmes against rabies in dogs (Cleaveland *et al*. 2003; WHO 2004; Schneider *et al*. 2005; Cleaveland *et al*. 2006; Davlin & VonVille 2012) and wildlife (Wandeler *et al*. 1988; Brochier *et al*. 1991; MacInnes *et al*. 2001; Rosatte *et al*. 2007) have proven efficacy and feasibility across a wide range of settings and raise far fewer ethical or welfare issues.
